# Association of Germline Variation in *CCNE1* and *CDK2* with Breast Cancer Risk, Progression and Survival among Chinese Han Women

**DOI:** 10.1371/journal.pone.0049296

**Published:** 2012-11-21

**Authors:** Ji-Yuan Han, Hui Wang, Yun-Tao Xie, Yan Li, Li-Yuan Zheng, Yuan Ruan, Ai-Ping Song, Xin-Xia Tian, Wei-Gang Fang

**Affiliations:** 1 Department of Pathology, Key Laboratory of Carcinogenesis and Translational Research (Ministry of Education), School of Basic Medical Sciences, Peking University Health Science Center, Beijing, People' Republic of China; 2 Breast Center, Peking University School of Oncology, Beijing Cancer Hospital & Institute, Beijing, People' Republic of China; Howard University, United States of America

## Abstract

**Background:**

Somatic alterations of cyclin-dependent kinase 2 (CDK2)-cyclin E complex have been shown to contribute to breast cancer (BC) development and progression. This study aimed to explore the effects of single nucleotide polymorphisms (SNPs) in *CDK2* and *CCNE1* (a gene encoding G1/S specific cyclin E1 protein, formerly called *cyclin E*) on BC risk, progression and survival in a Chinese Han population.

**Methodology/Principal Findings:**

We herein genotyped 6 haplotype-tagging SNPs (htSNPs) of *CCNE1* and 2 htSNPs of *CDK2* in 1207 BC cases and 1207 age-matched controls among Chinese Han women, and then reconstructed haplotype blocks according to our genotyping data and linkage disequilibrium status of these htSNPs. For *CCNE1*, the minor allele homozygotes of three htSNPs were associated with BC risk (rs3218035: adjusted odds ratio [aOR] = 3.35, 95% confidence interval [CI] = 1.69–6.67; rs3218038: aOR = 1.81, 95% CI = 1.22–2.70; rs3218042: aOR = 2.64, 95% CI = 1.31–5.34), and these three loci showed a dose-dependent manner in increasing BC risk (*P*_trend_ = 0.0001). Moreover, the 5-SNP haplotype CCGTC, which carried none of minor alleles of the 3 at-risk SNPs, was associated with a favorable event-free survival (hazard ratio [HR] = 0.53, 95% CI = 0.32–0.90). Stratified analysis suggested that the minor-allele homozygote carriers of rs3218038 had a worse event-free survival among patients with aggressive tumours (in tumour size>2 cm group: HR = 2.06, 95% CI = 1.06–3.99; in positive lymph node metastasis group: HR = 2.41, 95% CI = 1.15–5.03; in stage II–IV group: HR = 2.03, 95% CI = 1.09–3.79). For *CDK2*, no significant association was found.

**Conclusions/Significance:**

This study indicates that genetic variants in *CCNE1* may contribute to BC risk and survival in Chinese Han population. They may become molecular markers for individual evaluation of BC susceptibility and prognosis. Nevertheless, further validation studies are needed.

## Introduction

Breast cancer (BC) is the most common malignancy in women and annually causes 450 thousand deaths worldwide [1]. The research about genetic factors of BC has been a hot topic in decades. Several low-frequency, high-penetrance BC predisposition genes and low-frequency, intermediate-penetrance ones have been identified. The former includes *BRCA1, BRCA2, PTEN* and *p53*, and the latter involves *CHEK2, ATM, BRIP1* and *PALB2* [2]. Despite these discoveries, most of BC cannot be explained by the above genes. BC, as a common complex disease, may be interpreted by high-frequency, low-penetrance genetic variation according to the popular “common disease-common variants” hypothesis (CDCV) [3]. So far, SNPs, which amount to approximately 15 million in human genome [4], have become the most frequently used genetic markers in studying complex diseases. Through genome-wide association study (GWAS) and candidate gene strategy, some SNPs have been identified to be correlated with BC in different populations [5]–[7].

SNPs denote sites where the genomes of different people vary by a single base. A set of associated SNP alleles in a region of a chromosome is called a “haplotype”, while a pair of haplotypes forms a diplotype. Based on linkage disequilibrium (LD), which refers to the fact that particular alleles at nearby sites can co-occur on the same haplotype more often than is expected by accident in the genome [8], applying a minority of informative SNPs called haplotype-tagging SNPs (htSNPs) can capture the contribution of the whole gene to a specific phenotype [9]. Haplotype analysis involving htSNP genotyping is a cost-effective method when candidate gene strategy is adopted in population association study [10].

In cells, the cyclins and cyclin-dependent kinases (CDKs) interact at specific stages of the cell cycle to drive the cell cycle from one phase to the next. CDK2-cyclin E complex is known to initiate both DNA replication and centrosome duplication during the G1-S transition in the cell cycle [11]. Deregulated cyclin E induced chromosome instability (CIN) in human breast epithelial cells [12]. Two mechanisms that excess cyclin E induces CIN are put forward: one is defective S-phase progression, and the other is centrosome amplification [11]–[12]. Anomalies in cell-cycle control genes have frequently been observed in human malignancies including BC. The overexpression of *CCNE1* and high activity of CDK2-cyclin E are common in BC [11], [13]–[14]. Cyclin E has been found to be an important prognostic factor for patients with BC [15]–[17]. Amplification/overexpression of cyclin E has been suggested to be a mechanism of trastuzumab resistance in Her2 positive breast cancer patients [18] and an interaction between Her2 and cyclin E has been identified [19]. In addition, targeting cyclin E overexpression by siRNA could inhibit BC cell growth and suppress tumour development in BC mouse model [20]. Recently, a few association studies of genetic polymorphisms in cell cycle regulatory genes with risk or survival of some kinds of cancer have been reported [21]–[30]. They analyzed many potentially functional SNPs or tagging SNPs in cell cycle regulatory genes. However, for each of genes including *CCNE1* (a gene encoding cyclin E1 protein, formerly called *cyclin E*) and *CDK2*, they only evaluated the association of the selected individual SNPs or combination of them with risk or survival of cancers such as BC, lung cancer, endometrial cancer and ovary cancer [21]–[28], which couldn't capture the whole contribution of a gene to the development and progression of a particular cancer. In this study, we comprehensively analyzed the associations of htSNPs and haplotypes in *CCNE1* and *CDK2* with BC susceptibility, clinicopathological parameters and event-free survival in Chinese Han population,the largest ethnic group in China.

## Results

### Characteristics of the population

The selected characteristics of the cases and controls were summarized in Table 1. The cases and controls appeared to be adequately matched on age (*P* = 0.452). As expected, the BC cases had a younger age at menarche (*P*<0.0001) and an older age at first full-term pregnancy (*P*<0.0001) than controls. For other characteristics, such as body mass index (BMI), age at menopause, menopause status and family history of cancer in first-degree relatives, there was no statistical difference between cases and controls (*P*>0.05).

**Table 1:** Characteristics of BC cases and cancer-free controls.

Variable	Cases, n = 1207	Controls, n = 1207	*P*
Age, years (mean±SD)	48.98±10.07	48.68±9.85	0.452
BMI, mean (±SD)	24.58±3.12	24.48±3.55	0.441
Age at menarche, years (mean±SD)	14.54±1.81	15.03±1.88	<0.0001
Age at menopause, years (mean±SD)	49.01±4.16	49.15±3.97	0.593
Age at first full-term pregnancy, years (mean±SD)	26.13±2.98	25.40±2.74	<0.0001
Menopause status			0.102
Premenopause	630 (52.20%)	670 (55.51%)	
Postmenopause	577 (47.80%)	537 (44.49%)	
Family history of cancer in first-degree relatives			0.051
Yes	255 (21.13%)	217 (17.98%)	
No	952 (78.87%)	990 (82.02%)	
Estrogen receptor (ER)			
Positive	646 (53.52%)		
Negative	261 (21.62%)		
Missing data	300 (24.86%)		
Progesterone receptor (PR)			
Positive	597 (49.46%)		
Negative	306 (25.35%)		
Missing data	304 (25.19%)		
Her2			
Positive	241 (19.97%)		
Negative	663 (54.93%)		
Missing data	303 (25.10%)		
Tumor size in cm			
≤2 cm	391 (32.39%)		
>2 cm	538 (44.57%)		
Missing data	278 (23.04%)		
Lymph node metastasis			
Negative	467 (38.69%)		
Positive	337 (27.92%)		
Missing data	403 (33.39%)		
Clinical stage at diagnosis			
0–I	136 (11.27%)		
II–IV	692 (53.40%)		
Missing data	389 (32.23%)		

### LD degree between SNPs

**Figure 1:**
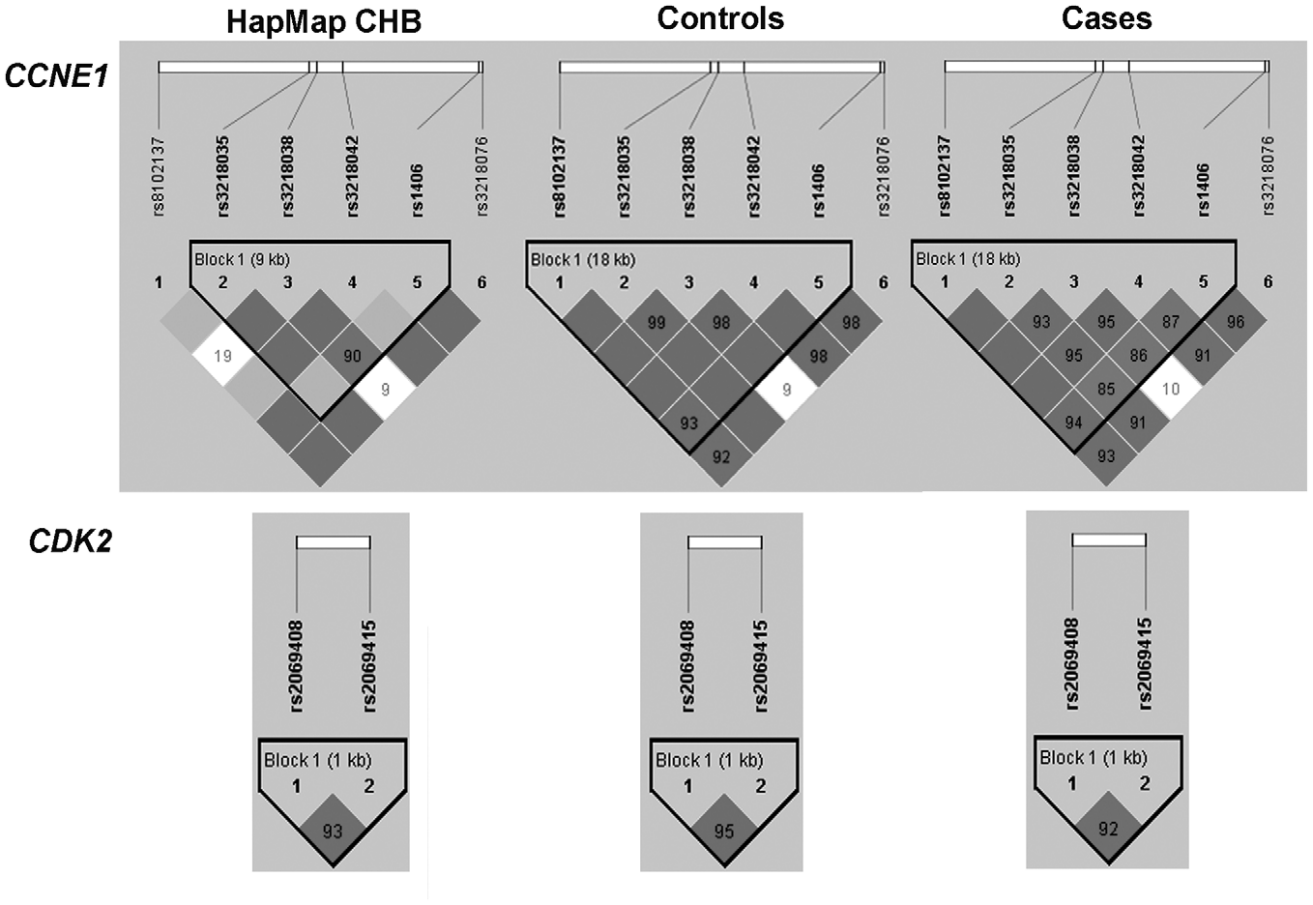
LD maps of eight htSNPs in HapMap CHB population, controls and BC cases. The values shown in each diamond are the D′ ×100 (10 means 0.10, 1 means 0.01). Dark grey diamonds without a number indicate that the value of D′ is 1. The dark grey-to-white gradient reflects higher to lower LD values.

The frequency distributions of genotypes and alleles for the eight SNPs among cases and controls were shown in Table 2. The eight SNPs were all in agreement with Hardy-Weinberg equilibrium (*P*>0.05) in the controls (data not shown). D′ and r^2^ between six SNPs in *CCNE1* and between two SNPs in *CDK2* within cases, controls and HapMap Han Chinese in Beijing (CHB) population were calculated using Haploview 4.2 software (Table S1). The LD degree of all SNPs in case population was consistent with that in control population (Figure 1). However, there were some differences between our control population and HapMap CHB population in the SNP genotyping data. The rs8102137 and rs3218038 were in strong LD in our control population (D′ = 1.000, r^2^ = 0.021), but in weak LD in HapMap CHB population (D′ = 0.191, r^2^ = 0.001). Therefore, we reconstructed a 5-SNP haplotype block (rs8102137, rs3218035, rs3218038, rs3218042 and rs1406) for *CCNE1* according to our genotyping data in cases and controls (Figure 1), while for *CDK2*, the 2-SNP haplotype block (rs2069408 and rs2069415) remained the same as in HapMap CHB population (Figure 1).

**Table 2:** Genotype and allele frequencies of the selected SNPs in *CCNE1* and *CDK2* and the association with risk of BC.

Gene	SNPs	Genotype	Cases (%)	Controls (%)	*P* *(*P* **)	*P* ***	*P*_trend_	OR (95% CI)	aOR (95% CI)†
*CCNE1*	rs8102137	TT	1016 (84.18)	1021 (84.59)	0.717 (0.694)		0.917		
		CT	183 (15.16)	175 (14.50)				1.05 (0.84–1.32)	1.01 (0.80–1.27)
		CC	8 (0.66)	11 (0.91)				0.73 (0.29–1.82)	0.67 (0.26–1.72)
		C allele frequency	0.082	0.082		0.917			
		CT/CC vs. TT (dominant model)						1.03 (0.83–1.29)	0.99 (0.79–1.24)
		CC vs. TT/CT (recessive model)						0.73 (0.29–1.81)	0.67 (0.26–1.72)
	rs3218035	CC	905 (74.85)	923 (76.47)	**0.002 (0.004)**		0.076		
		CT	267 (22.12)	273 (22.62)				1.00 (0.82–1.21)	1.01 (0.83–1.23)
		TT	35 (2.90)	11 (0.91)				**3.25 (1.64–6.43)**	**3.36 (1.69–6.70)**
		T allele frequency	0.139	0.122		0.078			
		CT/TT vs. CC (dominant model)						1.09 (0.90–1.31)	1.10 (0.91–1.33)
		TT vs. CC/CT (recessive model)						**3.25 (1.64–6.42)**	**3.35 (1.69–6.67)**
	rs3218038	GG	762 (63.13)	778 (64.46)	**0.015 (0.020)**		0.105		
		GT	374 (30.99)	388 (32.15)				0.98 (0.83–1.17)	1.01 (0.84–1.20)
		TT	71 (5.88)	41 (3.40)				**1.77 (1.19–2.63)**	**1.81 (1.21–2.71)**
		T allele frequency	0.214	0.195		0.101			
		GT/TT vs. GG (dominant model)						1.06 (0.90–1.25)	1.08 (0.91–1.28)
		TT vs. GG/GT (recessive model)						**1.78 (1.20–2.63)**	**1.81 (1.22–2.70)**
	rs3218042	TT	907 (75.14)	920 (76.22)	**0.016 (0.020)**		0.184		
		AT	271 (22.45)	276 (22.87)				1.00 (0.82–1.21)	1.01 (0.83–1.23)
		AA	29 (2.40)	11 (0.91)				**2.67 (1.33–5.38)**	**2.65 (1.31–5.36)**
		A allele frequency	0.136	0.123		0.214			
		AT/AA vs. TT (dominant model)						1.06 (0.88–1.28)	1.08 (0.89–1.30)
		AA vs. TT/AT (recessive model)						**2.67 (1.33–5.38)**	**2.64 (1.31–5.34)**
	rs1406	CC	529 (43.83)	519 (43.83)	0.350 (0.358)		0.787		
		AC	520 (43.08)	549 (45.48)				0.93 (0.78–1.10)	0.91 (0.77–1.09)
		AA	158 (13.09)	139 (11.52)				1.12 (0.86–1.44)	1.16 (0.89–1.52)
		A allele frequency	0.346	0.343		0.785			
		AC/AA vs. CC (dominant model)						0.97 (0.82–1.14)	0.96 (0.81–1.14)
		AA vs. CC/AC (recessive model)						1.16 (0.91–1.48)	1.22 (0.94–1.56)
	rs3218076	TT	421 (34.88)	401 (33.22)	0.075 (0.077)		0.728		
		GT	554 (45.90)	606 (50.21)				0.87 (0.73–1.04)	0.88 (0.73–1.06)
		GG	232 (19.22)	200 (16.57)				1.11 (0.88–1.40)	1.14 (0.90–1.44)
		G allele frequency	0.422	0.417		0.726			
		GT/GG vs. TT (dominant model)						0.93 (0.79–1.10)	0.94 (0.79–1.12)
		GG vs. TT/GT (recessive model)						1.20 (0.97–1.48)	1.22 (0.99–1.51)
*CDK2*	rs2069408	AA	669 (55.43)	660 (54.68)	0.636(0.643)		1.000		
		AG	451(37.37)	469 (38.86)				0.95 (0.80–1.12)	0.94 (0.79–1.12)
		GG	87(7.21)	78 (6.46)				1.10 (0.80–1.52)	1.15(0.82–1.61)
		G allele frequency	0.251	0.259		0.509			
		GG/AG vs. AA (dominant model)						0.97 (0.83–1.14)	0.97 (0.82–1.15)
		GG vs. AG/AA (recessive model)						1.12 (0.82–1.54)	1.18 (0.85–1.64)
	rs2069415	GG	884 (73.24)	867 (71.83)	0.440 (0.453)		0.662		
		AG	289 (23.94)	312 (25.85)				0.91 (0.76–1.09)	0.93 (0.77–1.13)
		AA	34 (2.82)	28(2.32)				1.19 (0.72–1.98)	1.26 (0.74–2.12)
		A allele frequency	0.148	0.152		0.658			
		AA/AG vs. GG(dominant model)						0.93 (0.78–1.11)	0.96 (0.80–1.16)
		AA vs. AG/GG(recessive model)						1.22 (0.74–2.02)	1.28(0.76–2.16)

† Adjusted for age, BMI, age at menarche, age at first full-term pregnancy, menopause status and family history of cancer in first-degree relatives.

* Two-sided χ2 test for difference in frequency distribution of genotypes between cases and controls.

** 1000 permutation tests for difference in frequency distribution of genotypes between cases and controls.

*** Two-sided χ2 test for difference in frequency distribution of alleles between cases and controls.

Bold numbers indicate a statistical significance at 0.05 level.

### Associations of genotypes, haplotypes and diplotypes with BC susceptibility

As shown in Table 2, two-sided χ^2^ test indicated no differences in allele frequencies between cases and controls for all eight SNPs, but showed significant differences in genotype frequencies of rs3218035, rs3218038 and rs3218042 in *CCNE1* (Table 2). Both univariate and multivariate unconditional logistic regression analyses showed that the minor allele homozygotes of rs3218035 (C>T), rs3218038 (G>T) and rs3218042 (T>A) could increase BC risk compared with heterozygotes and common homozygotes. To assess the relative importance of these three at-risk SNPs, we performed multiple logistic regression analyses including all 3 SNPs in the full model and used stepwise procedures to select the most important SNPs associated with BC risk. The result showed the OR value for rs3218035 increased marginally (OR = 3.93, 95% CI = 1.14–13.54, *P* = 0.031), whilst the statistical significance for rs3218038 and rs3218042 disappeared (rs3218038: OR = 1.50, 95% CI = 0.93–2.42, *P* = 0.099; rs3218042: OR = 0.58, 95% CI = 0.15–2.21, *P* = 0.426). We also examined the joint effects of these three at-risk loci on BC risk. Since r^2^ of rs3218035 and rs3218042 was 0.989 and 0.885 respectively in control and case populations, we regarded subjects carrying both at-risk loci of rs3218035 and rs3218042 as harboring one at-risk locus. As shown in Table 3, these at-risk loci showed a dose-dependent effect (*P*_trend_ = 0.0001).

**Table 3:** Risk of BC associated with the combination of 3 susceptible SNPs.

Genotype	Cases (%) n = 1207	Controls (%) n = 1207	OR (95% CI)	*P* value	aOR (95% CI)†	*P* value
Combinations of rs3218035 or rs3218042 with rs3218038‡						
0 risk loci	1127 (93.37)	1165 (96.52)				
1 risk loci	50 (4.14)	32 (2.65)	**1.62 (1.03–2.54)**	**0.037**	**1.70 (1.07–2.69)**	**0.024**
2 risk loci	30 (2.49)	10 (0.83)	**3.10 (1.51–6.37)**	**0.002**	**3.09 (1.49–6.38)**	**0.002**
*P*_trend_ = **0.0001**						

† Adjusted for age, BMI, age at menarche, age at first full-term pregnancy, menopause status and family history of cancer in first-degree relatives.

‡ Rs3218035 and rs3218042 can be tags for each other for r^2^ = 0.989 in the controls.

Risk loci are defined as homozygotes of minor allele of the 3 susceptible SNPs.

Bold numbers indicate a statistical significance at 0.05 level.

To better understand the contributions of the *CCNE1* and *CDK2* loci to BC development, we examined the associations between haplotypes in these two genes and BC risk. Neither the 5-SNP haplotypes in *CCNE1* nor the 2-SNP haplotypes in *CDK2* were associated with BC risk based on χ^2^ test and logistic regression analysis (Table S2). However, in *CCNE1*, the 5-SNP haplotype pairs (diplotype) TTTAC/TTTAC (rs8102137, rs3218035, rs3218038, rs3218042 and rs1406), which carried two minor alleles of 3 at-risk SNPs, rs3218035 (C>T), rs3218038 (G>T) and rs3218042 (T>A), could increase about 2.3-fold of BC risk compared with common diplotype TCGTC/TCGTA (OR = 2.35, 95% CI = 1.09–5.08, *P* = 0.029) (Table S3).

Then, we tested whether an interaction between genetic polymorphisms of *CCNE1* and *CDK2* may be associated with BC development. However, no significant interaction was found (data not shown).

### Associations of genotypes and haplotypes with BC clinicopathological parameters

Next, we analyzed the associations of genotype and haplotype with clinicopathological parameters, such as ER status, PR status, Her2 status, tumour size, lymph node status and clinical stage. We found that the patients with CT genotype of rs3218035 were more likely to have tumours with positive lymph node (OR = 1.47, 95% CI = 1.06–2.05, *P* = 0.022) (Table S4). Haplotype GG in *CDK2* was associated with stage II–IV tumours compared to common haplotype AG (OR = 1.73, 95% CI = 1.06–2.82, *P* = 0.027) (Table S5). No other significant association was observed.

### Associations of genotypes and haplotypes with event-free survival

**Figure 2:**
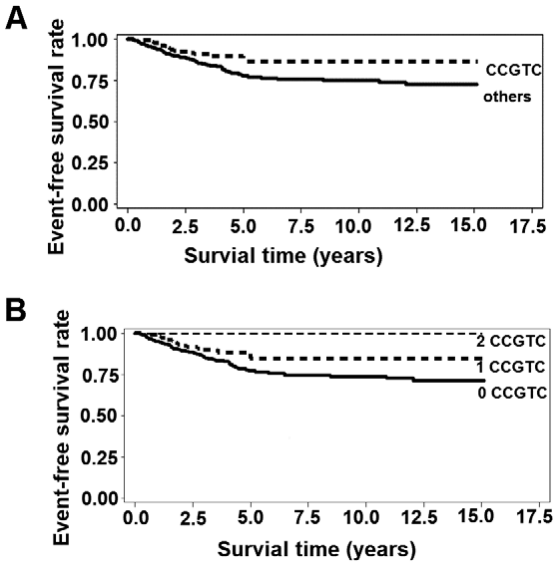
Kaplan–Meier estimates of event-free survival according to haplotype CCGTC.

**Figure 3:**
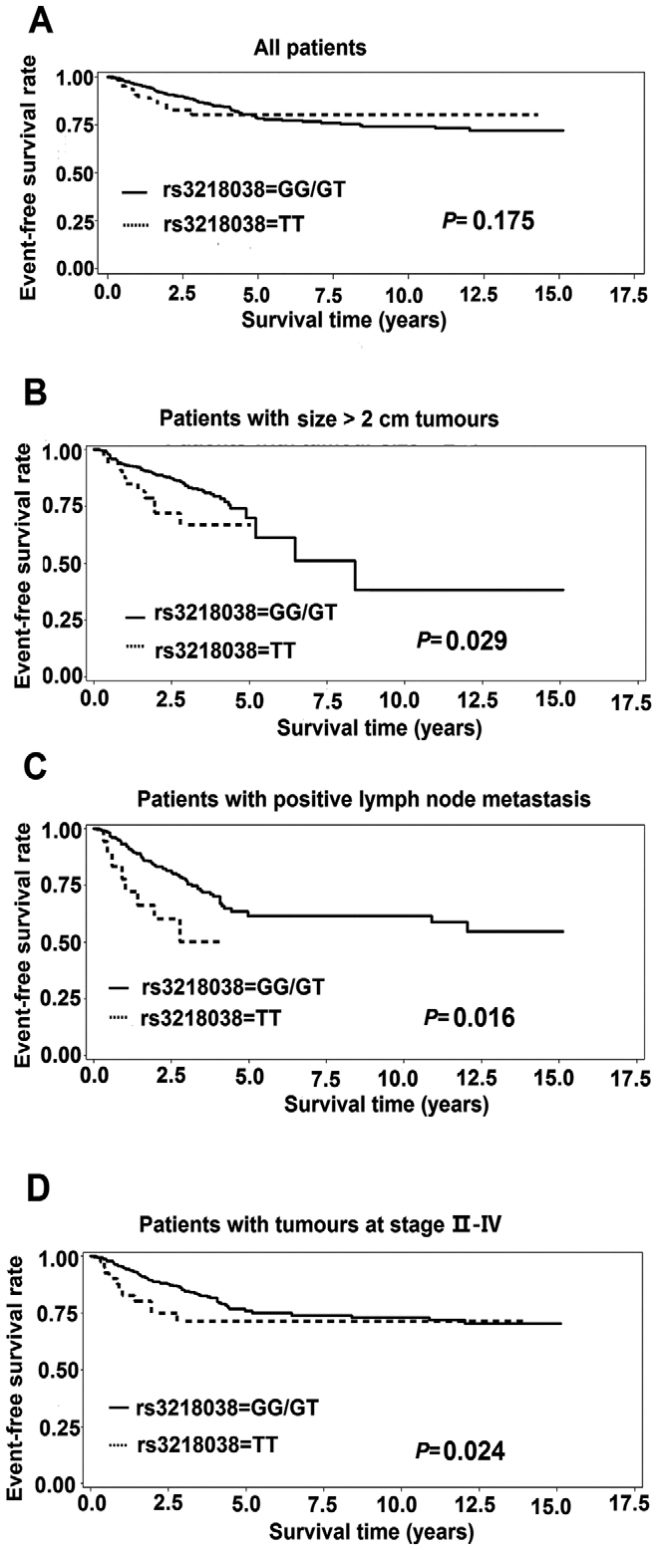
Kaplan–Meier estimates of event-free survival according to rs3218038 genotypes. **A** all BC patients; **B** patients with size >2 cm tumours; **C** patients with lymph node metastasis; **D** patients with tumours at stage II–IV. *P* values were calculated by log-rank test.

As we expected, aggressive clinicopathological parameters, such as negative PR status, positive Her2 status, tumour size>2 cm, lymph node metastasis and clinical stage II–IV, were associated with worse survival in the univariate Cox hazards regression analysis (Table 4). There was no association between individual SNPs and patients' survival (data not shown). However, haplotype CCGTC in *CCNE1* was correlated with a favorable event-free survival when compared to common haplotype TCGTC (HR = 0.53, 95% CI = 0.32–0.90, *P* = 0.018) or compared to all the other haplotypes (HR = 0.55, 95% CI = 0.33–0.91, *P* = 0.021) (Table 4). Notably, none of the six patients harboring homozygtes of haplotype CCGTC had BC-associated events during average 8-year follow up. The survival curves of CCGTC were shown in Figure 2. In addition, stratified analysis indicated TT-genotype carriers of rs3218038 (G>T) in *CCNE1* had unfavorable event-free survival compared with those carrying common G allele among patients with aggressive tumours (in tumour size>2 cm group: HR = 2.06, 95% CI = 1.06–3.99, *P* = 0.033; in positive lymph node metastasis group: HR = 2.41, 95% CI = 1.15–5.03, *P* = 0.019; in clinical stage II–IV group: HR = 2.03, 95% CI = 1.09–3.79, *P* = 0.027) (Table 5; Figure 3A–D). No other association with survival was observed.

**Table 4:** Univariate Cox proportional hazard analysis of the clinicopathological parameters and haplotypes in *CCNE1* and *CDK2* in relation to event-free survival of BC patients (n = 1007).

Parameter	No	No_event_ (%)	HR (95% CI)	*P* value
Age				
≤50 years	523	86 (16.44)		
>50 years	484	67 (13.84)	0.80 (0.58–1.10)	0.176
ER				
Positive	554	68 (12.27)		
Negative	229	37 (16.16)	1.32 (0.88–1.97)	0.183
PR				
Positive	514	58 (11.28)		
Negative	265	47 (17.74)	**1.66 (1.13–2.45)**	**0.010**
Her2				
Negative	577	68 (11.79)		
Positive	203	37 (18.23)	**1.52 (1.02–2.28)**	**0.040**
Lymph node status				
Negative	406	49 (12.07)		
Positive	280	82 (29.29)	**2.78 (1.95–3.97)**	**<0.0001**
Size				
≤2 cm	338	31 (9.17)		
>2 cm	466	84 (18.03)	**1.99 (1.32–3.01)**	**0.001**
Clinical stage				
0–I	113	9 (7.96)		
II–IV	582	107 (18.38)	**2.59 (1.31–5.11)**	**0.006**
*CCNE1* haplotypes				
TCGTC	712	116 (16.29)		
TCGTA	678	103 (15.19)	0.90 (0.69–1.17)	0.426
TTTAC	244	30 (9.80)	0.75 (0.50–1.12)	0.165
CCGTC	171	16 (9.36)	**0.53 (0.32–0.90)**	**0.018**
TCTTC	161	32 (10.46)	1.26 (0.85–1.87)	0.243
CCGTC vs all of others			**0.55 (0.33–0.91)**	**0.021**
*CDK2* haplotypes				
AG	1488	225 (15.12)		
GA	271	43 (15.87)	1.01 (0.73–1.41)	0.932
GG	240	36 (15.00)	0.99 (0.69–1.40)	0.940
AA	15	2 (13.33)	1.14 (0.28–4.59)	0.854

Bold numbers indicate a statistical significance at 0.05 level.

**Table 5:** Stratified event-free survival analysis of rs3218038 by lymph node status, tumor size, and clinical stage.

Variables	rs3218038 (G>T)	No.	No_event_ (%)	HR (95% CI)	*P* value	*P* for heterogeneity
All cases						
	GG+GT	947	141 (14.89)			
	TT	60	12 (20.00)	1.50 (0.83–2.70)	0.180	
Lymph node status						
Negative	GG+GT	380	45 (11.84)			0.99
	TT	26	4 (15.38)	1.35 (0.49–3.76)	0.562	
Positive	GG+GT	262	74 (28.24)			
	TT	18	8 (44.44)	**2.41 (1.15–5.03)**	**0.019**	
Size						
≤2 cm	GG+GT	319	31 (9.72)			0.406
	TT	19	0	-	-	
>2 cm	GG+GT	433	74 (17.09)			
	TT	33	10 (33.30)	**2.06 (1.06–3.99)**	**0.033**	
Clinical stage						
0–I	GG+GT	109	8 (7.34)			0.223
	TT	4	1 (25.00)	4.08 (0.50–33.29)	0.189	
II–IV	GG+GT	536	96 (17.91)			
	TT	38	11 (28.95)	**2.03 (1.09–3.79)**	**0.027**	

Bold numbers indicate a statistical significance at 0.05 level.

## Discussion

In the study, we evaluated the association of germline variation in *CCNE1* and *CDK2*, two essential cell cycle genes, with BC risk, progression and survival. To our knowledge, this is the first haplotype-based association study of *CCNE1* and *CDK2* with BC in Chinese Han population, which constitutes about 92% of the population of the People's Republic of China, and is the largest ethnic group in China and around the world.

For *CCNE1,* we analyzed 6 htSNPs, these being rs8102137 (T>C), rs3218035 (C>T), rs3218038 (G>T), rs3218042 (T>A), rs1406 (C>A) and rs3218076 (T>G), the first five of which was reconstructed as a 5-SNP haplotype block in our population. Three closely located SNPs, rs3218035, rs3218038 and rs3218042, were significantly associated with BC susceptibility under recessive models, and showed a dose-dependent effect (*P*_trend_ = 0.0001). The diplotype TTTAC/TTTAC (rs8102137, rs3218035, rs3218038, rs3218042 and rs1406), which carried two copies of minor alleles of the 3 at-risk SNPs, rs3218035 (C>T), rs3218038 (G>T) and rs3218042 (T>A), could increase about 2.3-fold of BC risk compared with common diplotype TCGTC/TCGTA. All of these demonstrated that SNPs could play a joint role in elevating BC risk. Stepwise procedure in logistic regression suggested rs3218035 was the leading contributor to BC risk among the three susceptible SNPs. Considering that cases with rare-allele homozygotes were too few to reach the statistical power for the 3 at-risk SNPs, we gave up further stratified analysis by environmental risk factors. The three susceptible SNPs are all located in intron 4, which may influence the disease risk by affecting mRNA expression levels, alternative splicing, mRNA structure and mRNA stability [31]–[32]. However, maybe they are only the tags of the causal variant. Fine-mapping to intron 4 and adjacent regions and further functional experiments are warranted. Functional analysis is a good way to determine whether one SNP is the causal variant. We plan to analyze the effects of at-risk SNPs in *CCNE1* on its mRNA and protein expression, and on cellular growth, centrosome amplification, DNA ploidy, transforming ability and so on. In survival analysis, a 5-SNP haplotype CCGTC, which carried no minor alleles of the 3 at-risk susceptible SNPs, was associated with a favorable event-free survival. Overall, the frequencies of CCGTC in nonaggressive tumour groups were higher than that in comparatively aggressive tumour groups, although all of the differences didn't reach statistical significance (CCGTC in Her2 negative group vs. Her2 positive group = 8.37% vs. 6.64%; size≤2 cm group vs. size>2 cm group = 9.08% vs. 7.34%; negative lymph node metastasis group vs. positive lymph node metastasis group = 8.89% vs. 7.42%; clinical stage 0–I group vs. clinical stage II–IV group = 10.66% vs. 7.44%) (Table S5). In stratified survival analysis, TT genotype of rs3218038 in *CCNE1* was associated with a worse event-free survival among patients with aggressive tumours (in tumour size>2 cm group: HR = 2.06, 95% CI = 1.06–3.99; in positive lymph node metastasis group: HR = 2.41, 95% CI = 1.15–5.03; in clinical stage II–IV group: HR = 2.03, 95% CI = 1.09–3.79). However, because of few cases with complete clinicopathological data, we didn't further perform prognostic factor-adjusted Cox regression analysis. Interestingly, Song H and colleagues genotyped 4 tag SNPs of *CCNE1* (rs997669, rs3218036, rs3218038 and rs3218076) in 1499 cases from the United Kingdom, Denmark and the United States, and found that rs3218038 had an effect on ovarian cancer survival (HR = 1.39, 95% CI = 1.04–1.85, *P* = 0.033) before adjusting for multiple hypothesis tests [28], which is consistent with our result. Therefore, rs3218038 deserves further exploration. Driver KE and colleagues examined 3 tag SNPs of *CCNE1* (rs997669, rs3218036 and rs3218076) in 4470 cases and 4560 controls from British population, and found that rs997669 in *CCNE1* was associated with BC risk (OR = 1.18, 95% CI = 1.04–1.34, *P* = 0.003) [25]. According to HapMap database, the minor allele frequency of rs997669 in the CEU (Utah residents with Northern and Western European ancestry from the CEPH collection) population is much higher than that in CHB population (36.3% vs. 5.8%). Its association with BC risk in Chinese population remains unclear. Azzato EM and colleagues analyzed 4 tag SNPs of *CCNE1* (rs997669, rs3218036, rs3218038 and rs3218076) in 4470 cases from England, and found no association between SNPs in *CCNE1* and BC survival [22]. The discrepancy in association of BC survival with rs3218038 between our data and those by Azatto EM et al could be explained as follows: First, we studied Chinese population, but Azzato EM studied Caucasian population; Second, we analyzed event-free survival and defined breast events such as BC recurrence/metastasis and death due to BC as the clinical endpoints. By contrast, Azzato EM analyzed overall survival and defined death due to any cause as the clinical endpoints. He also analyzed BC specific survival and defined death due to BC as the clinical endpoints; Third, although we found no association between rs3218038 and survival on the whole, we then further performed stratified analyses by tumour size, lymph node status and clinical stage and found rs3218038 was associated with a worse event-free survival among patients with aggressive tumours. However, Azzato EM did not carry out stratified analyses. Olson JE and colleagues genotyped 2 tag SNPs of *CCNE1* (rs997669 and rs1406) in 798 cases and 843 controls from the United States, and found no association between SNPs in *CCNE1* and BC risk [33]. In addition, rs8102137 was proved to be associated with bladder cancer risk in a multi-stage, genome-wide association study of European population [34]. The two SNPs with positive findings in our study, rs3218035 and rs3218042, were not studied in western population, because the minor allele frequency (MAF) of rs3218035 is 0.017, and rs3218042 is monopolymorphic in CEU population. Overall, these studies have controversial results, which could be due to the heterogeneity of populations, complicating environmental factors, different aetiologies of various cancers and the different roles of SNPs in development and progression of cancers.

For *CDK2*, we genotyped 2 htSNPs in our Chinese Han cohort, these being rs2069408 (A>G) and rs2069415 (G>A). These two htSNPs were in a single haplotype block in our population, and we performed individual SNPs and haplotype analyses. In this study, the single SNP, haplotype or haplotype pairs (diplotype) were not associated with BC risk or event-free survival. However, haplotype GG was more likely to be associated with clinical stage II–IV compared to the common haplotype AG. One research group genotyped 2 tag SNPs of *CDK2* (rs2069408 and rs1045435) in 4470 cases and 4560 controls from British population, and found no association of SNPs with BC risk and survival [22], [25]. There were also studies about the association of *CDK2* with ovarian cancer and endometrial cancer, and no significant association was observed [23], [26], [28].

In summary, 3 SNPs in *CCNE1*, rs3218035, rs3218038 and rs3218042, were identified to be associated with increased BC risk. The minor allele homozygote of rs3218038 in *CCNE1* was associated with a worse event-free survival among patients with aggressive tumours, and haplotype CCGTC was linked with a favorable event-free survival. Nevertheless, these genetic variants need to be investigated in other populations and verified by functional studies. More association studies on germline variants of other cell cycle regulatory genes such as *CDK4, CDK6, CDC2, cyclin D, cyclin A* and *cyclin B* would improve the ability of personalized evaluation of BC susceptibility and prognosis.

## Materials and Methods

### Study population

This population-based study is part of an ongoing cooperative study, the goal of which is to understand BC susceptibility and progression in Chinese Han women. This study included 1207 female BC patients and 1207 cancer-free female controls. All 1207 cases were pathologically diagnosed with primary infiltrating ductal carcinoma of the breast at the Beijing Cancer Hospital in China during the period 1995–2007. Their general information and clinicopathologic data were collected from the patients' medical records. The former included age at diagnosis, height, weight, age at menarche and/or menopause, menopause status, age at first full-term pregnancy and family history of cancer in first-degree relatives (parents, siblings and children). The latter involved ER status, PR status, Her2 status, tumour size, lymph node status and clinical stage based on the 6th edition of TNM staging of the American Joint Committee on Cancer (AJCC) system. For the cohort of cases, the last follow-up was performed on 31 August 2010. We used breast events including BC recurrence/metastasis and death due to BC as the clinical endpoints. The event-free survival time was calculated as the time from surgery to the occurrence of the study endpoints [35]. Censoring events included death by a cause other than BC, voluntarily withdrawing from the study and lack of a significant breast event before 31 August 2010. The median follow-up time after surgery was 3.4 years. Of the 1207 cases, 48 cases had no operation, 132 were lost to follow-up and 20 died of unknown cause. Thus, there remained 1007 cases in the event-free survival analysis.

The 1207 controls were selected from cancer-free women participating in a community-based screening programme for non-infectious diseases conducted in Beijing, China. The selection criteria included no history of cancer, Chinese Han ethnic background and age-matched to cases (same 5-year group). All eligible controls completed an epidemiological questionnaire.

This study was approved by the Peking University IRB (reference no. IRB00001052-11029). Written consents were obtained from all control samples. BC samples were collected initially for research purposes in the tissue/blood biobank. Written consents were collected from the BC patients who can read and write. Verbal consents were obtained from the BC patients who cannot read and write, however, for these cases, written consent was signed by her next of kin. The IRB approved the written consent procedure. The data/samples were used anonymously. PKU IRB approved our application to waive informed re-consent for the already collected BC samples in the tissue/blood biobank. This study only used this part of samples.

### SNPs Selection

All SNPs in *CCNE1* and *CDK2* genes were selected according to the public HapMap database (HapMap Data Release #27; Chinese Beijing population) and the NCBI dbSNP database (dbSNP b126; Chinese Beijing population). For *CCNE1* gene, 21 common SNPs, minor allele frequency (MAF) >5%, were identified and two high-LD blocks were constructed by the Haploview programme, spanning from 10 kb upstream of the transcriptional start site to 10 kb downstream of the 3′ UTR. Six haplotype-tagging SNPs (htSNPs) within two LD blocks were selected by Haploview software 4.2 [36], these being rs8102137 in the 5′ franking region, rs3218035, rs3218038 and rs3218042 in the intron 4, rs1406 in the 3′UTR, and rs3218076 in the 3′ franking region. In *CDK2* gene, only 2 common SNPs in a single LD block were identified in CHB population according to HapMap database, these being rs2069408 in intron 5 and rs2069415 in the 3′UTR.

### Genotyping assays and quality control

Genomic DNA was isolated from blood leukocytes by proteinase K digestion followed by phenol–chloroform extraction and isopropanol precipitation. Genotyping was carried out by using Taqman Assay® (Applied Biosystems) according to manufacturer's instructions. Primers and FAM- and VIC- labeled probes were supplied directly by Applied Biosystems as Assays-by-Design™ or Assays-on-Demand™ products. All assays were performed by using the ABI Step One® Real-Time PCR System (Applied Biosystems, FosterCity, California). The PCR conditions were the same as that described earlier by Yuan Ruan and colleagues [37]. At least 1% of samples were duplicated randomly in each SNP assay, and the concordance between duplicates was more than 99%.

### LD block determination and haplotype construction

Pairwise measures of LD measured by Lewontin coefficient (D′) and squared correlation coefficient (r^2^) between the genotyped SNPs were calculated, and then haplotype blocks in cases and controls were reconstructed respectively with the Haploview 4.2 software. For each participant, the most probable haplotypes were estimated using the SAS9.1 PROC HAPLOTYPE procedure according to expectation – maximization (EM) algorithm.

### Statistical analysis

Differences in demographic characteristics and selected variables between cases and controls were compared by two-sided chi-square (χ^2^) test (for categorical variables) or student's *t* test (for continuous variables). For each SNP, Hardy–Weinberg equilibrium in control subjects was examined by a one-degree-of-freedom goodness-of-fit test. A two-sided χ^2^ test was used to compare differences in the distributions of genotypes and alleles between cases and controls, and to evaluate associations of genotypes and haplotypes with clinicopathological parameters. A permutation procedure (1000 tests) was carried out to correct the *P* value in the individual SNP analysis. To determine the effect of the genetic polymorphisms on BC risk, odds ratios (ORs) and 95% confidence intervals (95% CIs) were calculated in univariate and multivariate unconditional logistic regression models, without and with adjustment for age, body mass index (BMI), age at menarche, menopause status, age at first full-term pregnancy and family history of cancer in first-degree relatives [37]–[38]. Each genotype was assessed according to codominant, dominant and recessive models [39]. The survival curves were derived using Kaplan–Meier method, and verified by the log-rank test. To further investigate the associations of clinicopathological parameters, genotypes and haplotypes with event-free survival, hazard ratio (HR) and 95% CIs were calculated using univariate Cox proportional hazards model. All statistic analyses were done with Statistic Analysis System software (v.9.1; SAS Institute, Cary, NC).

## Supporting Information

Table S1 **D′and r^2^ between pairs of htSNPs in** ***CCNE1*** **and** ***CDK2*** **among cases, controls and HapMap CHB population.**
(DOC)

Table S2 **Haplotype frequencies of the** ***CCNE1*** **and** ***CDK2*** **genes in 1207 cases and 1207 controls and the association with risk of BC.**
(DOC)

Table S3 **Diplotype frequencies of** ***CCNE1*** **in 1207 cases and 1207 controls and the association with risk of BC.**
(DOC)

Table S4 **Associations of genotypes in** ***CCNE1*** **and** ***CDK2*** **with clinicopathological parameters.**
(DOC)

Table S5 **Associations of haplotypes in** ***CCNE1*** **and** ***CDK2*** **with clinicopathological parameters.**
(DOC)
