# Supplementary material for: Association of Germline Variation in CCNE1 and CDK2 with Breast Cancer Risk, Progression and Survival among Chinese Han Women
Source: PLoS One. 2012 Nov 21;7(11):e49296. doi: 10.1371/journal.pone.0049296 (PMC3504019; doi:10.1371/journal.pone.0049296)
Supplement: Table S1 — D′and r2 between pairs of htSNPs in CCNE1 and CDK2 among cases, controls and HapMap CHB population. (DOC) [file pone.0049296.s001.doc]

**Table S1. D’and r^2^ between pairs of htSNPs in *CCNE1* and *CDK2* among cases, controls and HapMap CHB population**

| SNP pairs | | D' Cases/controls/HapMap | r^2^ Cases/controls/HapMap |
| --- | --- | --- | --- |
| rs8102137 | rs3218035 | 1.000/1.000/1.000 | 0.015/0.012/0.013 |
| rs8102137 | rs3218038 | 1.000/1.000/0.191 | 0.024/0.021/0.001 |
| rs8102137 | rs3218042 | 1.000/1.000/1.000 | 0.014/0.013/0.013 |
| rs8102137 | rs1406 | 0.942/0.933/1.000 | 0.042/0.040/0.063 |
| rs8102137 | rs3218076 | 0.937/0.926/1.000 | 0.058/0.054/0.100 |
| rs3218035 | rs3218038 | 0.935/0.991/1.000 | 0.521/0.565/0.359 |
| rs3218035 | rs3218042 | 0.954/1.000/1.000 | 0.885/0.989/1.000 |
| rs3218035 | rs1406 | 0.852/1.000/1.000 | 0.062/0.073/0.072 |
| rs3218035 | rs3218076 | 0.919/1.000/1.000 | 0.100/0.099/0.114 |
| rs3218038 | rs3218042 | 0.954/0.986/1.000 | 0.528/0.567/0.359 |
| rs3218038 | rs1406 | 0.866/1.000/0.909 | 0.108/0.126/0.171 |
| rs3218038 | rs3218076 | 0.106/0.093/0.093 | 0.0020/0.001/0.003 |
| rs3218042 | rs1406 | 0.873/1.000/1.000 | 0.064/0.073/0.072 |
| rs3218042 | rs3218076 | 0.914/0.984/1.000 | 0.096/0.097/0.114 |
| rs1406 | rs3218076 | 0.960/0.986/1.000 | 0.669/0.710/0.629 |
| rs2069408 | rs2069415 | 0.923/0.954/1.000 | 0.623/0.469/0.425 |
