# Supplementary material for: Association of Germline Variation in CCNE1 and CDK2 with Breast Cancer Risk, Progression and Survival among Chinese Han Women
Source: PLoS One. 2012 Nov 21;7(11):e49296. doi: 10.1371/journal.pone.0049296 (PMC3504019; doi:10.1371/journal.pone.0049296)
Supplement: Table S2 — Haplotype frequencies of the CCNE1 and CDK2 genes in 1207 cases and 1207 controls and the association with risk of BC. (DOC) [file pone.0049296.s002.doc]

**Table S2. Haplotype frequencies of the *CCNE1* and *CDK2* genes** in 1207 cases and 1207 controls and the association with risk of BC

| Gene | Haplotype | Cases (%) | Controls (%) | OR (95% CI) | *P* value | aOR (95% CI) † | *P* value |
| --- | --- | --- | --- | --- | --- | --- | --- |
| *CCNE1* | rs8102137 (T>C)+rs3218035 (C>T)+rs3218038 (G>T)+rs3218042 (T>A)+rs1406 (C>A) | | | | | | |
|  | TCGTC | 861 (35.67) | 918 (38.03) |  |  |  |  |
|  | TCGTA | 817 (33.84) | 825 (34.18) | 1.06 (0.92-1.21) | 0.427 | 1.07 (0.93-1.23) | 0.343 |
|  | TTTAC | 303 (12.55) | 292 (12.10) | 1.11 (0.92-1.33) | 0.286 | 1.15 (0.95-1.39) | 0.162 |
|  | CCGTC | 197 (8.16) | 195 (8.08) | 1.08 (0.87-1.34) | 0.506 | 1.05 (0.84-1.31) | 0.679 |
|  | TCTTC | 183 (7.58) | 175 (7.25) | 1.12 (0.89-1.40) | 0.348 | 1.16 (0.92-1.47) | 0.219 |
|  | Others | 53 (2.20) | 9 (0.36) | - | - | - | - |
| *CDK2* | rs2069408 (A>G)+rs2069415 (G>A) | | | | | | |
|  | AG | 1771 (73.36) | 1778 (73.65) |  |  |  |  |
|  | GA | 339 (14.04) | 357 (14.79) | 0.95 (0.81-1.12) | 0.564 | 0.99 (0.83-1.16) | 0.855 |
|  | GG | 286 (11.85) | 268 (11.10) | 1.07 (0.90-1.28) | 0.451 | 1.07 (0.89-1.28) | 0.473 |
|  | AA | 18 (0.75) | 11 (0.46) | - | - | - | - |

†Adjusted for age, BMI, age at menarche, age at first full-term pregnancy, menopause status and family history of cancer in first-degree relatives.
