# Supplementary material for: Association of Germline Variation in CCNE1 and CDK2 with Breast Cancer Risk, Progression and Survival among Chinese Han Women
Source: PLoS One. 2012 Nov 21;7(11):e49296. doi: 10.1371/journal.pone.0049296 (PMC3504019; doi:10.1371/journal.pone.0049296)
Supplement: Table S3 — Diplotype frequencies of CCNE1 in 1207 cases and 1207 controls and the association with risk of BC. (DOC) [file pone.0049296.s003.doc]

**Table S3. Diplotype frequencies of *CCNE1* in 1207 cases and 1207 controls and the association with risk of BC**

| Diplotype | Cases (%) | Controls (%) | OR (95% CI) | *P* value | aOR (95% CI) † | *P* value |
| --- | --- | --- | --- | --- | --- | --- |
| TCGTC/TCGTA | 289 (23.94) | 324 (26.84) |  |  |  |  |
| TCGTC/TCGTC | 165 (13.67) | 171 (14.17) | 1.08 (0.83-1.41) | 0.563 | 1.09 (0.83-1.44) | 0.541 |
| TCGTA/TCGTA | 150 (12.43) | 137 (11.35) | 1.23 (0.93-1.63) | 0.778 | 1.29 (0.97-1.73) | 0.085 |
| TCGTC/TTTAC | 102 (8.45) | 115 (9.53) | 0.99 (0.73-1.36) | 0.972 | 1.04 (0.75-1.43) | 0.831 |
| TCGTA/TTTAC | 92 (7.62) | 108 (8.95) | 0.96 (0.69-1.32) | 0.778 | 0.99 (0.71-1.39) | 0.967 |
| TCGTA/CCGTC | 61 (5.05) | 66 (5.47) | 1.04 (0.71-1.52) | 0.856 | 0.97 (0.65-1.43) | 0.865 |
| TCGTC/CCGTC | 77 (6.38) | 65 (5.39) | 1.33 (0.92-1.92) | 0.129 | 1.27 (0.87-1.86) | 0.211 |
| TCGTA/TCTTC | 68 (5.63) | 49 (4.06) | 1.56 (1.04-2.32) | 0.030 | 1.55 (1.03-2.34) | 0.038 |
| TCGTC/TCTTC | 59 (4.89) | 69 (5.72) | 0.96 (0.65-1.41) | 0.828 | 0.97 (0.65-1.43) | 0.875 |
| CCGTC/TTTAC | 32 (2.65) | 24 (1.99) | 1.50 (0.86-2.60) | 0.154 | 1.53 (0.87-2.71) | 0.140 |
| TCTTC/TTTAC | 27 (2.24) | 23 (1.91) | 1.32 (0.74-2.35) | 0.352 | 1.45 (0.80-2.65) | 0.225 |
| TTTAC/TTTAC | 21 (1.74) | 10 (0.83) | **2.35 (1.09-5.08)** | **0.029** | **2.42 (1.11-5.28)** | **0.026** |
| Others | 64 (5.30) | 46 (3.81) | - | - | - | - |
| TCGTC/TCGTA | 289 (23.94) | 324 (26.84) |  |  |  |  |
| Any other haplotype/TTTAC | 261 (21.62) | 272 (22.54) | 1.10 (0.87-1.39) | 0.423 | 1.13 (0.89-1.44) | 0.302 |
| TTTAC/TTTAC | 21 (1.74) | 10 (0.83) | **2.35 (1.09-5.08)** | **0.029** | **2.42 (1.11-5.28)** | **0.026** |
| *P*_trend_=0.094 | | | | | | |

†Adjusted for age, BMI, age at menarche, age at first full-term pregnancy, menopause status and family history of cancer in first-degree relatives.

Bold numbers indicate a statistical significance at 0.05 level.
