# Supplementary material for: Association of Germline Variation in CCNE1 and CDK2 with Breast Cancer Risk, Progression and Survival among Chinese Han Women
Source: PLoS One. 2012 Nov 21;7(11):e49296. doi: 10.1371/journal.pone.0049296 (PMC3504019; doi:10.1371/journal.pone.0049296)
Supplement: Table S4 — Associations of genotypes in CCNE1 and CDK2 with clinicopathological parameters. (DOC) [file pone.0049296.s004.doc]

**Table S4. Associations of genotypes in *CCNE1* and *CDK2* with clinicopathological parameters**

| Polymorphisms | ER |  |  | PR |  |  | Her2 |  |  | Lymph node status | |  | Size |  |  | Clinical stage | |  |
| --- | --- | --- | --- | --- | --- | --- | --- | --- | --- | --- | --- | --- | --- | --- | --- | --- | --- | --- |
|  | P† | N‡ | *P* value* | P† | N‡ | *P* value* | N‡ | P† | *P* value* | N‡ | P† | *P* value* | ≤2 cm | >2 cm | *P* value* | 0-Ⅰ | Ⅱ-Ⅳ | *P* value* |
| rs8102137 (T>C) |  |  |  |  |  |  |  |  |  |  |  |  |  |  |  |  |  |  |
| TT | 548 | 218 | 0.623 | 509 | 254 | 0.376 | 553 | 211 | 0.128 | 388 | 288 | 0.364 | 321 | 70 | 0.139 | 109 | 593 | 0.100 |
| CT+CC | 98 | 43 |  | 88 | 52 |  | 110 | 30 |  | 79 | 49 |  | 70 | 77 |  | 27 | 99 |  |
| rs3218035 (C>T) |  |  |  |  |  |  |  |  |  |  |  |  |  |  |  |  |  |  |
| CC | 476 | 194 | 0.854 | 439 | 227 | 0.767 | 487 | 184 | 0.630 | 359 | 239 | **0.040**** | 289 | 405 | 0.349 | 107 | 511 | 0.49 |
| CT | 121 | 87 |  | 137 | 71 |  | 154 | 51 |  | 94 | 92 |  | 86 | 120 |  | 26 | 164 |  |
| TT | 22 | 7 |  | 21 | 8 |  | 22 | 6 |  | 14 | 6 |  | 16 | 13 |  | 3 | 17 |  |
| rs3218038 (G>T) |  |  |  |  |  |  |  |  |  |  |  |  |  |  |  |  |  |  |
| GG | 401 | 162 | 0.850 | 365 | 194 | 0.695 | 411 | 152 | 0.619 | 300 | 202 | 0.386 | 239 | 347 | 0.518 | 96 | 425 | 0.127 |
| GT | 199 | 83 |  | 192 | 90 |  | 204 | 76 |  | 138 | 115 |  | 127 | 156 |  | 34 | 226 |  |
| TT | 46 | 16 |  | 40 | 22 |  | 48 | 13 |  | 29 | 20 |  | 25 | 35 |  | 6 | 41 |  |
| rs3218042 (T>A) | |  |  |  |  |  |  |  |  |  |  |  |  |  |  |  |  |  |
| TT | 475 | 194 | 0.944 | 440 | 225 | 0.697 | 490 | 180 | 0.907 | 358 | 240 | 0.129 | 293 | 403 | 0.990 | 109 | 510 | 0.286 |
| AT | 154 | 61 |  | 140 | 75 |  | 156 | 56 |  | 98 | 91 |  | 88 | 122 |  | 25 | 168 |  |
| AA | 17 | 6 |  | 17 | 6 |  | 17 | 5 |  | 11 | 6 |  | 10 | 13 |  | 2 | 14 |  |
| rs1406 (C>A) |  |  |  |  |  |  |  |  |  |  |  |  |  |  |  |  |  |  |
| CC | 292 | 119 | 0.820 | 269 | 142 | 0.658 | 305 | 105 | 0.462 | 208 | 151 | 0.221 | 176 | 246 | 0.757 | 57 | 303 | 0.819 |
| AC | 270 | 112 |  | 249 | 130 |  | 271 | 109 |  | 210 | 138 |  | 165 | 216 |  | 60 | 305 |  |
| AA | 84 | 30 |  | 79 | 34 |  | 87 | 27 |  | 49 | 48 |  | 50 | 76 |  | 19 | 84 |  |
| rs3218076 (T>G) |  |  |  |  |  |  |  |  |  |  |  |  |  |  |  |  |  |  |
| TT | 232 | 93 | 0.979 | 210 | 115 | 0.766 | 243 | 81 | 0.415 | 164 | 117 | 0.979 | 143 | 195 | 0.686 | 47 | 235 | 0.943 |
| GT | 290 | 119 |  | 271 | 135 |  | 289 | 117 |  | 221 | 159 |  | 166 | 241 |  | 63 | 331 |  |
| GG | 124 | 49 |  | 116 | 56 |  | 131 | 43 |  | 82 | 61 |  | 82 | 102 |  | 26 | 126 |  |
| rs2069408(A>G) | |  |  |  |  |  |  |  |  |  |  |  |  |  |  |  |  |  |
| A/A | 367 | 151 | 0.923 | 340 | 176 | 0.438 | 336 | 150 | 0.087 | 262 | 195 | 0.394 | 219 | 300 | 0.858 | 81 | 387 | 0.493 |
| A/G | 230 | 92 |  | 217 | 103 |  | 248 | 71 |  | 167 | 123 |  | 141 | 200 |  | 49 | 257 |  |
| G/G | 49 | 18 |  | 40 | 27 |  | 49 | 20 |  | 38 | 19 |  | 31 | 38 |  | 6 | 48 |  |
| rs2069415(G>A) | |  |  |  |  |  |  |  |  |  |  |  |  |  |  |  |  |  |
| G/G | 477 | 198 | 0.782 | 443 | 230 | 0.854 | 487 | 185 | 0.243 | 333 | 254 | 0.058 | 291 | 396 | 0.719 | 100 | 509 | 0.632 |
| A/G | 150 | 55 |  | 137 | 66 |  | 157 | 46 |  | 116 | 79 |  | 87 | 128 |  | 31 | 167 |  |
| A/A | 19 | 8 |  | 17 | 10 |  | 19 | 10 |  | 18 | 4 |  | 13 | 14 |  | 5 | 16 |  |

†P= positive; ‡N= negative; *Two-sided χ2 test; **Statistically significant (P<0.05); CT compared to CC in logistic regression: OR = 1.47, 95% CI = 1.06-2.47, *P* = 0.022.
