# Supplementary material for: Association of Germline Variation in CCNE1 and CDK2 with Breast Cancer Risk, Progression and Survival among Chinese Han Women
Source: PLoS One. 2012 Nov 21;7(11):e49296. doi: 10.1371/journal.pone.0049296 (PMC3504019; doi:10.1371/journal.pone.0049296)
Supplement: Table S5 — Associations of haplotypes in CCNE1 and CDK2 with clinicopathological parameters. (DOC) [file pone.0049296.s005.doc]

**Table S5. Associations of haplotypes of *CCNE1* and *CDK2* with** clinicopathological parameters

| Haplotype | ER |  |  | PR |  |  | Her2 |  |  | Lymph node status | |  | Size |  |  | Stage |  |  |
| --- | --- | --- | --- | --- | --- | --- | --- | --- | --- | --- | --- | --- | --- | --- | --- | --- | --- | --- |
|  | P† | N‡ | *P* value* | P† | N‡ | *P* value* | N‡ | P† | *P* value* | N‡ | P† | *P* value* | ≤2 cm | >2 cm | *P* value* | 0-Ⅰ | Ⅱ-Ⅳ | *P* value* |
| TCGTC | 35.45 | 36.78 | 0.59 | 35.09 | 37.42 | 0.33 | 35.29 | 37.97 | 0.30 | 36.72 | 35.31 | 0.56 | 34.02 | 37.45 | 0.13 | 36.03 | 35.98 | 0.99 |
| TCGTA | 33.36 | 30.84 | 0.30 | 33.58 | 30.39 | 0.17 | 32.50 | 32.99 | 0.85 | 32.44 | 33.68 | 0.60 | 32.61 | 33.46 | 0.70 | 36.03 | 33.60 | 0.44 |
| TTTAC | 13.62 | 11.88 | 0.32 | 13.74 | 12.09 | 0.33 | 13.20 | 12.03 | 0.51 | 12.10 | 14.39 | 0.18 | 12.53 | 12.73 | 0.90 | 10.66 | 13.15 | 0.26 |
| CCGTC | 7.82 | 8.24 | 0.76 | 7.62 | 8.50 | 0.51 | 8.37 | 6.64 | 0.23 | 8.89 | 7.42 | 0.29 | 9.08 | 7.34 | 0.18 | 10.66 | 7.44 | 0.07 |
| TCTTC | 7.59 | 8.62 | 0.46 | 7.96 | 7.84 | 0.93 | 7.84 | 8.30 | 0.75 | 7.82 | 7.57 | 0.85 | 8.06 | 7.34 | 0.57 | 5.51 | 8.02 | 0.15 |
| Others | 2.17 | 3.64 | - | 2.01 | 3.76 | - | 2.79 | 2.07 | - | 2.03 | 1.63 | - | 3.71 | 1.67 | - | 1.10 | 1.81 | - |
| Overall |  |  | 0.36 |  |  | 0.16 |  |  | 0.67 |  |  | 0.64 |  |  | 0.05 |  |  | 0.22 |
| AG | 73.61 | 74.90 | 0.57 | 74.20 | 73.53 | 0.76 | 73.15 | 75.73 | 0.27 | 72.70 | 75.67 | 0.18 | 72.76 | 73.70 | 0.65 | 77.57 | 73.55 | 0.17 |
| GA | 13.54 | 13.03 | 0.77 | 13.40 | 13.24 | 0.92 | 13.95 | 12.45 | 0.41 | 14.99 | 12.46 | 0.15 | 13.17 | 13.85 | 0.67 | 15.07 | 13.44 | 0.47 |
| GG | 11.84 | 11.49 | 0.84 | 11.47 | 12.42 | 0.56 | 12.14 | 10.58 | 0.36 | 11.03 | 11.42 | 0.80 | 12.79 | 11.80 | 0.52 | 7.35 | 12.07 | **0.03**** |
| AA | 1.01 | 0.58 | 0.37 | 0.92 | 0.82 | 0.82 | 0.75 | 1.24 | 0.33 | 1.28 | 0.45 | 0.08 | 1.28 | 0.65 | 0.16 | 0.00 | 0.94 | 0.11 |
| Overall |  |  | 0.81 |  |  | 0.94 |  |  | 0.46 |  |  | 0.15 |  |  | 0.47 |  |  | 0.05 |

†P= positive; ‡N= negative; *Two-sided χ2 test; each haplotype compared with all other haplotypes ;**Statistically significant (*P*<0.05); GG compared to AG in logistic regression: OR = 1.73, 95% CI = 1.06-2.82, *P* = 0.027.
